# Pseudo‐mechanistic Explanations in Psychology and Cognitive Neuroscience

**DOI:** 10.1111/tops.12448

**Published:** 2019-07-29

**Authors:** Bernhard Hommel

**Affiliations:** ^1^ Institute for Psychological Research & Leiden Institute for Brain and Cognition Leiden University

**Keywords:** Cognitive mechanisms, Causal explanations, Cognitive neuroscience, Circular explanation, Galilean psychology

## Abstract

Few articles in psychology and cognitive neuroscience do without the promise to get into the “mechanisms underlying” particular psychological phenomena. And yet the progress in our mechanistic understanding of human cognition and behavior must be considered disappointing: Most “explanations” merely classify the phenomenon under investigation as falling into a broader category of (not any better understood) phenomena, specify the context conditions under which the phenomenon is likely to occur, or specify a particular kind of neural activity (such as the activation of a particular brain area) that is correlated with the phenomenon. None of these meets the criteria of a truly mechanistic explanation, which needs to account for phenomena in terms of “a structure performing a function in virtue of its component parts, component operations, and their organization” (Bechtel, 2006). This contribution characterizes the problem and some of its implications and discusses possible solutions.

## Abstract 2

Pseudo‐mechanistic explanations in psychology and cognitive neuroscience

This paper focuses on the level of systems/cognitive neuroscience. It argues that the great majority of explanations in psychology and cognitive neuroscience is “pseudo‐mechanistic.” On the basis of various case studies, Hommel argues that cognitive neuroscience should move beyond what he calls an “Aristotelian phase” to become a mature “Galilean” science seeking to discover actual mechanisms of cognitive phenomena.

## 1 Introduction

Psychologists and cognitive (neuro)scientists try to explain human behavior by unraveling the (functional^1^ and neural) mechanisms underlying it. But what counts as a mechanistic explanation? According to Cummins (2010), the primary explanandum of psychology are (human) *capacities*, such as our ability to perceive depth, to learn things, to act voluntarily, etc. To provide a mechanistic account of a capacity, theorists need to describe how it emerges from the interplay of more basic elements: “A mechanism is a structure performing a function in virtue of its component parts, component operations, and their organization. The orchestrated functioning of the mechanism is responsible for one or more phenomena” (Bechtel, 2006, p. 26). For parts of a to‐be‐explained system to contribute to the explanation, they must have a stable set of properties, must be robustly detectable, and should be open to interventions (Craver, 2006). For instance, in order to explain an automobile’s capacity to move, one would need the basic concepts of an engine (transforming input energy into movement), a transmission system (translating movement into movements of other parts), and a wheel (the rotation of which moves the object when contacting a surface), and the basic idea of how these components interact to move the automobile. A suitable theory thus presupposes some basic understanding of the functional role of each part in the organization and of the way this role is played. Such a theory could be said to capture the essence of what an automobile is, irrespective of the specifics, so that it could be equally applied to automobiles that differ in input energy (petrol, gas, electricity), architecture of the engine, kind of transmission, and shape and number of wheels. Translated into cognitive (neuro)science, a good mechanistic theory would thus consist of a clear *specification of its components*, such as the codes or representations of the relevant informational units, *and of the organization of these components*, including the processes operating on them (Bechtel, 2008, 2009). In other words, *mechanistic theories need to explain how structures relate to processes, and vice versa*.

Clearly, neuroscientific theorizing calls for different ways to identify and characterize the organized components and processes than cognitive theorizing does: It is specific to neural implementation, where components might range from single neurons to entire brain structures and processes from synchronized spiking to changes in connectivity. In contrast, cognitive theorizing is agnostic with respect to the neural implementation but rather focuses on the functional or informational aspects and implications of representational units and the processes that orchestrate their activation states and interactions. However, while some theoreticians seem to lean toward neural reductionism (e.g., Bechtel, 2008) or at least consider neural underpinnings as more fundamental and mechanistic than functional underpinnings, there is no reason to believe that functional theorizing cannot be as mechanistic (in precisely specifying how structures relate to processes, and vice versa) as neural theorizing can be. For instance, there is no sense in which an account of human imitation in terms of mirror neurons (Rizzolatti & Craighero, 2004) is necessarily any more mechanistic, fundamental, or specific than functional accounts that attribute imitation to the feature‐overlap of stimulus and response representations (Hommel et al., 2001). Along the same lines, there is also no sense in which neural and functional theories would necessarily differ systematically with respect to grain size or hierarchical relationship; for example, many functional connectionist models are targeting representations and processes at a much lower level than neural theories regarding functional differences of cortical hemispheres. Accordingly, nothing prevents us from applying the same criteria for judging the mechanistic character of neural and functional theories.

It is tempting to relate neural and functional theorizing to the levels of theorizing advocated by Marr (1982, Marr & Poggio, 1976), whose *implementational* level is arguably the target of what I consider neural theorizing while his *representational/algorithmic* level, “which specifies the forms of the representations and the algorithms defined over them” (McClamrock, 1991), is the target of functional theorizing. As I have pointed out, theorizing at both of these levels should be considered mechanistic only, and to the degree that it explains how structures relate to processes, and vice versa. One can doubt that this applies to Marr’s third, the *computational* level that calls for a task analysis (what task is a system carrying out?), as the adequacy of task analyses is commonly judged by their heuristic power rather than their mechanistic stringency.

The main concern that I would like to voice is the fact that few theories in cognitive (neuro)science can be considered mechanistic according to the suggested criteria^2^ and, worse, very little effort is being spent on developing theories that do—suggesting that the absence of mechanistic theorizing is not even considered a problem. As I will elaborate below, some functional theories specify processes, sometimes even computationally, without providing an idea of the components on which these processes operate, while others provide representational details without specifying the processes operating on them. Similarly, some neural theories specify neural substrates thought to contribute to a particular phenomenon without providing an idea of what these substrates do, why it might be these substrates that do it, and how their interaction is orchestrated. But why are theorists so unwilling to provide all the ingredients required to build a mechanistic model? Here I suggest that this may have to do with the fact that cognitive (neuro)science is still in an early phase of development, a phase that Lewin (1931) has characterized as Aristotelian (as compared to Galilean).^3^ The defining characteristic of Aristotelian theorizing is the assumption that sorting observations into theoretically defined categories is sufficient to explain them, and it may be this assumption that stands in the way of building truly mechanistic models.

## 2 Aristotelian and Galilean psychology

According to Lewin (1931), Aristotelian psychology is characterized by a category‐based top–down approach to study psychological processes, in which psychological concepts are taken from everyday life observations and turned into “valuative” and binary categories (“normal”/“pathological”, “true perception”/“illusion”). To explain a novel observation, the researcher needs to assign it to the fitting category, not much different from biology, where identifying a newly discovered animal as a member of an existing species provides all the explanation one needs. The major scientific ambition is restricted to observations that are highly consistent and replicable, while variability is considered unlawful and thus falling outside of the task of science. The focus is rather on group means, which are considered to capture the essence of the natural laws laying behind and explaining the observation.

According to Hempel and Oppenheim (1948), this research strategy amounts to “explanation as subsumption under natural law." In physics, this strategy enjoys widespread popularity, as it for instance allows deriving simpler laws for movements of particular objects from Newton’s law of motion. However, psychological researchers equate actual natural laws with categories of observations (Cummins, 2010)—a practice that Fiedler (1991) has coined “empirical generalization." Similarly, the theorizing in cognitive neuroscience is commonly restricted to attributing a particular observation to the activation of either a particular brain system or network—which is then assumed to be sufficient to explain this observation. The key problem with this Aristotelian understanding‐by‐categorizing approach is that it does not provide insight into the actual mechanism. This is sometimes easy to recognize by the circularity of the explanation, such as when the observation that some stimuli attract more attention than others is “explained” by their “salience” (Theeuwes, 2010)—their potency to attract attention, and the ability to put oneself into the shoes of others by having a right temporal parietal junction (Saxe, Carey, & Kanwisher, 2004)—a brain system that somehow does it.

An Aristotelian research strategy may be unavoidable in the infancy of a scientific discipline, but it distracts from the eventual goal of understanding human capacities and fails to provide any mechanistic insight. This helps researchers to organize available observations into a category system that reflects the characteristics of the tasks generating them, but it remains entirely unclear whether these characteristics bear any relationship with the lawful processes underlying the capacities that await mechanistic understanding. The Aristotelian approach thus fosters paradigm‐driven research, in which the theoretical ambitions of the researchers are limited to re‐describing the available findings in a modeling language. For instance, decades of research on human memory has mainly engaged in sorting memory‐related behavior into an ever‐increasing number of categories assumed to reflect corresponding memory systems, without much progress in our mechanistic understanding of how processes need to operate on codes to generate the observed behavior (Bechtel, 2008)—only to arrive at the possible conclusion that memory processes are so much integrated with other cognitive activities that a dedicated memory system may actually not exist (Buckner & Schacter, 2004).

But what is the alternative? The Galilean research strategy that Lewin (1931) contrasts with the Aristotelian strategy holds promise to provide such an alternative (Hommel & Colzato, 2015). It differs from the Aristotelian by (1) not respecting binary distinctions into categories but rather trying to account for all available findings in terms of gradations or degrees of expression of one common principle (e.g., “normal” and “pathological” behavior would need to be explained through the same mechanism); (2) not taking pre‐scientific categories from everyday language and analyzing them into multiple subcomponents but rather starting with a well‐understood basic mechanism and trying to account for as many observations as possible; and (3) considering inter‐ and intra‐individual variability not as measurement noise but as observations that good mechanistic theory needs to account for. The heuristic power of these three choices consists in the fact that they make the lack of mechanistic thinking in Aristotelian sorting practices particularly obvious: Once researchers need to do more than just assigning binary labels to observe phenomena, but also need to account for variations of the phenomenon, individual variability therein, and mechanistic overlap with other, similar phenomena, it becomes clear that mere sorting doesn’t do.

Lewin’s plea for a transition from Aristotelian to Galilean psychology was published in the 1930s, when psychology was still a developing discipline. One may thus wonder whether his characterization of the everyday practice as Aristotelian still holds. Do psychologists and cognitive neuroscientists still categorize rather than explain? In the following, I will briefly discuss representative examples from five research domains, the first three from behavioral psychology with more functional explanatory goals and two more from the cognitive neurosciences. I would like to emphasize that these are just examples that could be easily replaced by others, so the cases that I did pick should not be taken as more representative of Aristotelian thinking than others.

## 3 Stimulus‐response compatibility

Since the 1950s, there is increasing interest in observations suggesting an apparently privileged (compatible) relationship between some stimuli and some responses, which were difficult to explain in terms of the then‐popular information‐processing kind of theorizing: For example, people can press a left key faster if being signaled with a left than a right stimulus, and name the color of a word faster if the task‐irrelevant meaning of the word is congruent with the color—the notorious Stroop effect. One of the key questions that these observations are posing is why the task‐irrelevant stimulus information is processed up to a degree that can even activate the corresponding response (Hommel, 2011).

As true for many phenomena investigated in the 1970s and later, effects of this kind were investigated by means of the Sternberg (1969) logic, according to which the presence or absence of interactions between independent variables can be systematically used to identify the processing stage a particular phenomenon is “located." The major aim of theorizing was thus to decide at which processing stage phenomena like stimulus‐response compatibility are “located.” and successful localization (at the “response selection stage” for compatibility phenomena) was considered to be sufficient for explaining the phenomenon. Note the absence of any ambition to identify the details of what might be going on at a given stage, be it regarding the codes/representations being processed or the processes operating on them.

An example is the most comprehensive model of stimulus‐response compatibility suggested by Kornblum, Hasbroucq, and Osman (1990). The authors argue that compatibility effects can be categorized according to particular congruency relationships between the relevant and the irrelevant aspect of stimulus and response, which leaves the authors with five (later eight) categories. This theoretical category system serves to reduce explanation to categorization, just as Lewin’s concept of Aristotelian research describes: Whenever a novel observation is made under conditions that fit with the category system (i.e., with particular stimulus‐response combinations), the observation is thought to be sufficiently understood and theoretically explained. The mechanistic question of *why* and *how* the irrelevant stimulus aspect is translated into response activation is not explained but built into the model, which simply assumes *that* it does. This, however, was previously demonstrated empirically, which renders the account an uninformative re‐description of available findings.

A more Galilean alternative was provided by Hommel et al. (2001). Rather than a dedicated model of stimulus‐response compatibility, they provided a general theory of human perception and action planning, which produces stimulus‐response compatibility phenomena as one of many byproducts. In particular, the theory describes both the components (representations of stimuli and responses) and the processes operating on these components to generate cognitive phenomena, and it has been implemented in a computational framework that demonstrates how the representational components emerge ontogenetically through experience (Haazebroek, Raffone, & Hommel, 2017). Other extensions have shown that the framework accounts for both basic effects and individual variability (Hommel & Wiers, 2017).

## 4 Psychological refractory period

In 1931, Telford observed that performance in speeded reaction time tasks declines as the time between trials decreases, which suggested to him that the process of response selection might be easy to overload if being used too often during a particular time interval. Later studies have extended these observations by systematically varying the time between tasks of different kinds and a complex (“locus‐of‐slack”) methodology was developed to attribute the corresponding effects to particular processing stages. Hundreds of studies have been conducted by using this methodology, with the main outcome being that Telford was right: Response selection suffers from temporal overload (Pashler, 1994). The research practice in this area is a perfect example for the Aristotelian sorting strategy: The goal of the research consists in categorizing the effect of a given independent manipulation by assigning it to a hypothetical processing stage, for which no further theoretical justification exists (apart from common‐sense considerations: see Sternberg, 1969).

Researchers in the field have apparently accepted this categorization as providing sufficient insight into the phenomenon, as no efforts have been undertaken so far to provide a mechanistic account that could explain *why* and because of which processing characteristics response selection is more sensitive to overload than other stages. This is particularly surprising as theoretically considering the codes/representations and the processes involved provide obvious options. As speculated elsewhere (Hommel, 1998), the eventual selection of a response under conditions in which multiple action plans are concurrently active creates two problems: a *binding problem*, as the representational codes of more than one action are activated, and an *order problem*, as the standard instruction in dual‐task experiments requires sequential performance. Both problems go beyond the information available to the system or stage responsible for response selection, as they call for the integration of stimulus information, response information, and the appropriate stimulus‐response mapping. This involves almost all cognitive stages/systems busy with the task and integration of information across the entire cognitive system (or brain)—suggesting that response selection in multitasking situations may face the by far highest demands with respect to both information integration and to‐be‐covered neural distance. Characterizing and understanding the mechanisms underlying these demands requires moving from current sorting practices to getting to grips with task representations and the processes orchestrating them.

## 5 Thinking

Theorizing about human thinking represents a particularly obvious example for Aristotelian sorting. Despite differences in detail, the general idea is that thinking proceeds along two routes or systems: a rational/conscious route/system that generates solutions that fit with normative models of human rationality and an irrational/unconscious route/system that accounts for the rest (e.g., Evans, 2003). The theoretical strategy is obvious: Empirical observations are sorted into two categories, often by using a not further justified normative model, and then two hypothetical systems are conceived that have no other purpose and no other function than producing exactly these observations. Successful categorization is then considered to be sufficient to explain the categorized behavior.

The normative basis of dual‐system theorizing has been criticized. For instance, Goldstein and Gigerenzer (2002) have argued that decisions based on non‐logical thinking does not need to be incorrect but may often enjoy high ecological validity—for example, guessing that a city with a more familiar name might be larger will often be successful. Computational models specifying algorithms that describe which and how environmental cues are processed to inform decision‐making have been suggested (see ABC Research Group, 2006), even though the representational architecture on which these algorithms operate, the origin, nature, and characteristics of the codes that store the relevant environmental information, and the mechanisms integrating information from different sensory modalities, are still underspecified. Possible improvements have been suggested by Schooler and Hertwig (2005), who integrate Gigerenzer’s ecological approach with ACT‐R, a computational architecture with specific assumptions regarding representations and cognitive‐processing operations (Anderson & Lebiere, 1998). A similar step toward more Galilean alternatives has been taken by Cleeremans and Jiménez (2002), who offer a computational framework that provides a functional description of both the algorithms responsible for generating “thoughts” (i.e., outcomes of decision‐making processes) and the representations on which these algorithms operate.

## 6 Theory of mind

This term tries to capture the fascinating ability to take other people’s minds, and the contents thereof, into account. Cognitive neuroscience approaches account for this ability by assuming a hypothetical “mentalizing system” (Amodio & Frith, 2006; overview in Overwalle & Baetens, 2009), which is thought to comprise the cortical midline structures and the temporoparietal junction (TPJ). As typical for neuroscientific approaches, the contributions made by these components are determined by correlating the activity of the respective brain area with particular tasks. For instance, the right TPJ has been frequently shown to be active in tasks that require predicting other people’s actions in situations where oneself has other, commonly more information about some state of affairs than the to‐be‐predicted person—such as when a sought‐for object has been relocated after this person has left the room. These correlations have led researchers (such as Rebecca Saxe at her TED talk on “How we read each other’s mind” in 2009) to claim that having a right TPJ is sufficient to explain the human capacity to read other people’s minds.

Claims of that sort have been criticized for various reasons: Jumping from correlation (between task and TPJ activity) to causality requires experimental manipulations of TPJ functioning (e.g., by means of brain stimulation), and mind‐reading may involve only subparts of TPJ. But what concerns me here is rather the idea that having a brain area can ever be a sufficient mechanistic explanation for a psychological capacity. Obviously, assuming that brain activity can tell us something about mental capacities relies on some materialist/functionalist agreement that psychological processes and brain activity are two sides of the same coin, irrespective of how complex the relationship might be. This makes it trivial to show that engaging in a particular psychological process activates parts of the brain. Such activity would only be of interest if the involvement of TPJ would have particular implications: It may receive particular kinds of input, produce a particular kind of output, exhibit a particular processing style, or have particular structural characteristics that may inform us about the actual mechanism. Without all that information, the mere equation of mind‐reading and TPJ goes nowhere beyond Aristotelian sorting, which makes no contribution to something that could count as a mechanistic explanation of *how* we generate insights into other people’s minds.

## 7 Imitation

People can imitate the behavior of others, which provides enormous advantages for learners and the transmission of cultural knowledge, but we still do not know how this is possible. Research has generated a good understanding of how the seeing of someone performing a particular dance figure, say, is (functionally and neurally) coded by an observer and how she would actively perform this figure herself. What remains unclear, however, is how the distributed coding of features in dedicated maps of the visual cortex eventually activates the muscles that successfully re‐create exactly those movements: How can seeing be systematically translated into acting without ever having done this before? Given the very different coding principles in visual and motor cortex (Prinz, 1992), this is not a trivial task, which raises the question how the translation is achieved. Cognitive neuroscience is widely believed to have provided the answer: mirror neurons or the “mirror system” (Rizzolatti & Craighero, 2004). Notwithstanding criticism regarding some details of this approach (e.g., regarding whether/how single‐neuron recordings in monkeys relate to fMRI findings in humans, causality, or the specific coding format), having mirror neurons is generally assumed to provide a sufficient explanation of how people can imitate.

But is it? Accepting the identity of mind and brain necessarily implies that, if humans can connect what they perceive to what they do, as when they imitate, there need to be neurons that represent this connection. This means that that the existence of mirror neurons (i.e., neurons that are active in both perception and production of a dancing figure) is no hypothesis with any empirical content but a necessity. Of course, necessity does not predict where those neurons are located, which input they receive, which computations they are involved in, and which output they produce, so that the discovery of mirror neurons is no doubt a great scientific achievement. And yet, this discovery makes no contribution to the mechanistic explanation of the human capacity to imitate. In that sense, accounts that accept the mere existence of mirror neurons as a sufficient explanation must be considered to represent Aristotelian thinking.

Galilean solutions are again possible: Keysers and Perrett (2004) have suggested the basis for a computational framework, in which the components of a mirror system and their organization are specified. Interestingly, the orchestration of this system can produce imitation as just one byproduct, but it can also help understanding how people plan and perform intentional actions; in fact, the framework can be considered to represent the first neuro‐computational approach to ideomotor theory (see Hommel, 2009).

## 8 Conclusion

The take‐home message from this contribution is that no truly mechanistic explanation is provided by assigning an empirical observation to a particular functional system or linking it to the activation of a particular brain area. If so, many theoretical accounts in cognitive (neuro)science must be considered pseudo‐mechanistic and a reflection of Aristotelian logic. Truly mechanistic accounts, I have argued, require the specification of the components that a given mechanism comprises of, and of the processes that organize these components to generate the phenomenon under investigation. As I tried to show, the accounts that meet these criteria are rare and their absence is commonly not even missed.

Cognitive and neurocognitive theory thus needs to become more ambitious in terms of aims and mechanistic detail—irrespective of whether the explanatory language is functional, neural, or computational in nature. Explanations need to go beyond postulating a hypothetical system that has no further purpose than just producing the observations one aims to explain, and beyond considering neural correlates of an observation an explanation. Encouraging researchers to become more Galilean in thinking and practice is likely to require changes in the mindsets of reviewers and editors, who would need to learn appreciating truly mechanistic approaches that do not aim to explain effects of particular experimental paradigms but, rather, account for general human capacities. Very likely, this would be the end of paradigm‐driven research and the beginning of cross‐paradigmatic theorizing, which I consider the next stage of the maturation process of our discipline.

## Notes

1 This terminology implies that true understanding of cognitive mechanism requires the eventual integration of functional and neural descriptions, as both provide important constraints for each other. I acknowledge that this does not fit, and is not supposed to fit, with the terminology in some more philosophical papers (e.g., Weiskopf, 2011), where “functional” descriptions are contrasted with truly mechanistic descriptions, implying that functional descriptions are mechanistically inferior.

2 Note that my focus on the mechanistic adequacy of explanations in psychology/cognitive neurosciences is not to deny other possible flaws of psychological explanations, such as the possible cultural dependency of many phenomena (Gergen, 1973), the incompleteness of processing models (Newell, 1973), and the tautological nature of many theories (Wallach & Wallach, 1998), but the lack of mechanistic ambition that I criticize here cannot be fully reduced to any of these factors.

3 Note that my aim is not to justify the use of the terms “Aristotelian” and “Galilean” in a historical‐philosophical sense; I simply take them as a label to distinguish two approaches to psychology.
